# Adverse events associated with nicotine replacement therapy (NRT) for smoking cessation. A systematic review and meta-analysis of one hundred and twenty studies involving 177,390 individuals

**DOI:** 10.1186/1617-9625-8-8

**Published:** 2010-07-13

**Authors:** Edward J Mills, Ping Wu, Ian Lockhart, Kumanan Wilson, Jon O Ebbert

**Affiliations:** 1 Faculty of Health Sciences, University of Ottawa, Ottawa, Canada; 2 Department of Epidemiology, London School of Hygiene & Tropical Medicine, London, UK; 3 Pfizer Limited, Tadworth, Surrey, UK; 4 Ottawa Health Research Institute, Ottawa, Canada; 5 Mayo Graduate School of Medicine, Mayo Clinic, Rochester, MN, USA

## Abstract

**Background:**

Nicotine replacement therapy (NRT) is the most common form of smoking cessation pharmacotherapy and has proven efficacy for the treatment of tobacco dependence. Although expectations of mild adverse effects have been observed to be independent predictors of reduced motivation to use NRT, adverse effects associated with NRT have not been precisely quantified.

**Objective:**

A systematic review and meta-analysis aimed to identify all randomized clinical trials (RCTs) of NRT versus inert controls and all observational studies to determine the magnitude of reported adverse effects with NRT.

**Methods:**

Searches of 10 electronic databases from inception to November 2009 were conducted. Study selection and data extraction were carried out independently in duplicate. RCTs were pooled using a random effects method with Odds Ratio [OR] as the effect measure, while proportions were pooled from observational studies. A meta-regression analysis was applied to examine whether the nicotine patch is associated with different adverse effects from those common to orally administered NRT.

**Results:**

Ninety-two RCTs involving 32,185 participants and 28 observational studies involving 145, 205 participants were identified. Pooled RCT evidence of varying NRT formulations found an increased risk of heart palpitations and chest pains (OR 2.06, 95% Confidence Interval [CI] 1.51-2.82, P < 0.001); nausea and vomiting (OR 1.67, 95% CI 1.37-2.04, P < 0.001); gastrointestinal complaints (OR 1.54, 95% CI, 1.25-1.89, P < 0.001); and insomnia (OR 1.42, 95% CI, 1.21-1.66, P < 0.001). Pooled evidence specific to the NRT patch found an increase in skin irritations (OR 2.80, 95% CO, 2.28-3.24, P < 0.001). Orally administered NRT was associated with mouth and throat soreness (OR 1.87, 95% CI, 1.36-2.57, P < 0.001); mouth ulcers (OR 1.49, 95% CI, 1.05-2.20, P < 0.001); hiccoughs (OR 7.68, 95% CI, 4.59-12.85, P < 0.001) and coughing (OR 2.89, 95% CI, 1.92-4.33, P < 0.001). There was no statistically significant increase in anxiety or depressive symptoms associated with NRT use. Non-comparative observational studies demonstrated the prevalence of these events in a broad population.

**Conclusion:**

The use of NRT is associated with a variety of side effects. In addition to counseling and medical monitoring, clinicians should inform patients of potential side effects which are associated with the use of NRT for the treatment of tobacco dependence.

## Introduction

Smoking is the leading cause of preventable mortality world wide[1]. One in every 2 long-term smokers will die a smoking related death[2]. Stopping smoking has a considerable impact on improving life expectancy, reducing morbidity and reducing health care costs associated with treating smoking related conditions[3].

Several pharmacological interventions to assist in smoking cessation are available[4]. The most commonly used formulation is nicotine replacement therapy (NRT), frequently available over the counter (OTC). NRT is currently recommended as a safe intervention to general populations and higher-risk groups, including pregnant and breastfeeding women, adolescents, and smokers with cardiovascular disease[5]. NRT improves cessation rates at one year by approximately 70% (odds ratio [OR] 1.70, 95% Confidence Interval [CI] 1.55-1.88)[4,6].

Available research suggests that smokers are less motivated to use NRT if they expect that it will cause mild adverse effects[7]. Published systematic reviews of NRT have not explicitly synthesized the incidence of side effects of NRT products. An understanding of the nature and likelihood of the most common side effects may help clinicians communicate to patients the benefits and risks associated with their use of NRT. This information may also improve selection of specific delivery mechanisms based upon patient history, which may improve treatment adherence. While RCTs provide strong information on causation of adverse events, observational studies may report on associations or hypotheses about more rare events. To determine the frequency and incidence of adverse events associated with NRT, we conducted a systematic review and meta-analysis of RCT's and observational studies of NRT in any delivery formulation. Our clinical question is, in patients receiving NRT for smoking cessation, compared to inert controls, what is the incidence of adverse events and what are those adverse events?

## Methods

### Eligibility criteria

We included RCTs of any duration beyond 4 weeks. RCTs had to compare NRT with an inert control (eg. placebo or standard of care). We chose 4 weeks to include the timeframe of maximum nicotine withdrawal symptoms so that adverse events may be differentiated from withdrawal symptoms[8]. We additionally sought out observational studies to examine the proportion of events occurring[9]. We evaluated adverse effects reported at any point in the duration of the studies. We included any form of NRT delivery (i.e. lozenge, skin patch, gum, nasal spray, inhaler, and tablet). We did not examine efficacy in this analysis. We excluded post-hoc analyses, maintenance therapy, or relapse prevention studies.

### Search strategy

In consultation with a medical librarian, we established a search strategy. We searched independently, in duplicate, the following 10 databases (from inception to November 20, 2009): MEDLINE, EMBASE, Cochrane CENTRAL, AMED, CINAHL, TOXNET, Development and Reproductive Toxicology, Hazardous Substances Databank, Psych-info and Web of Science. Given that observational studies are poorly indexed in many databases, we also searched databases that include the full text of journals (*ScienceDirect*, and *Ingenta*, including articles in full text from approximately 1700 journals since 1993)[10]. In addition, we searched the bibliographies of published systematic reviews and health technology assessments[4-6,11-16]. Searches were not limited by language, sex or age.

### Study selection

Two investigators (EM, PW) working independently, in duplicate, scanned all abstracts and obtained the full text reports of records indicating that the study was either an RCT or observational study evaluating NRT on the outcomes of interest. After obtaining full reports of the candidate studies (either in full peer-reviewed publication or press article) the same reviewers independently assessed eligibility via full text review.

### Data collection

Two reviewers (EM, PW) conducted data extraction independently using a standardized pre-piloted form. Reviewers collected information about the NRT intervention tested, the population studied (age, sex, underlying conditions), treatment dosages and dosing schedules, the specific measurement of abstinence (prolonged or point-prevalence), and the methods of biochemical confirmation. The reviewers extracted data on adverse events characterized by the study authors as physical or mental adverse events. Recognizing that adverse events may include both physical and mental effects concomitantly, we defined physical adverse events as effects confined to physical parts of the body and mental adverse events as symptoms accompanied by psychological conditions. We characterized serious adverse events as unexpected life-threatening events occurring during the trial period. *A priori*, we examined the follow life-threatening adverse events: all-cause mortality, myocardial infarction, all-cause strokes, incidence of all-cancers, all-hospitalizations, suicidal ideation, depression, and incidence of diabetes. Study quality evaluation included general methodological reporting features including allocation concealment, sequence generation, blinding status, intention-to-treat, and appropriate descriptions of loss to follow-up. In rating quality, failure to report a quality component of study design (e.g. blinding) was treated the same as not employing it. We entered the data into an electronic database such that duplicate entries existed for each study. When the two entries did not match, we resolved differences through discussion and consensus. In the absence of an inert control group, we considered randomized NRT dosing studies to be observational studies and collected data on proportion of combined events. For cohort studies, we additionally calculated events as proportions of events.

### Data analysis

In order to assess inter-rater reliability on inclusion of articles, we calculated the *Phi *statistic (*ϕ*), which provides a measure of inter-observer agreement independent of chance[17]. We calculated the Odds Ratios [OR] and appropriate 95% Confidence Intervals [CIs] of outcomes according to the number of events reported in the original studies or sub-studies. Given that zero events in one treatment arm prevents a useful ratio from being developed, in circumstances of zero outcome events in one arm of a trial, we added 0.5 to each arm, as suggested by Sheehe[18]. We first pooled studies of all NRT interventions versus all controls using the DerSimonian-Laird random effects method[19], which recognizes and anchors studies as a sample of all potential studies, and incorporates an additional between-study component to the estimate of variability[20]. We calculated the I^2^ statistic for each analysis as a measure of the proportion of the overall variation that is attributable to between-study heterogeneity[21]. Given that we are examining adverse events, interpreting heterogeneity estimates can be challenging as even pooled analysis with large heterogeneity may provide important insights into the likelihood of events[9]. We considered an I^2 ^above 50% as moderate to large heterogeneity and examined explanations of heterogeneity by applying a random effects meta-regression with the following co-variates: oropharyngeal formulation vs. skin patch; duration of study (in months); reporting of allocation concealment, and reporting of blinding status. We then calculated the residual heterogeneity and present it as the residual I^2^. Forest plots are displayed for each primary analysis, showing pooled study effect measures with 95% CIs, and the overall DerSimonian-Laird pooled estimate. For studies considered as observational studies, we calculated pooled weighted proportions by first stabilizing the variances of the raw proportions (r/n) using a Freeman-Tukey type arcsine square root transformation and applying a random effects model. While several methods of pooling proportions exist, the Freeman-Tukey method works well with both fixed and random effects meta-analysis and truncates at zero[22]. This is a variance-stabilizing transformation that removes the dependence of the variance on the mean of the transformed proportion (ie. it corrects for overdispersion). Assessing heterogeneity in pooled proportions may be misleading[23,24], therefore we report the I^2 ^value, as this measure is less affected by the number of studies as the more commonly used I^2^. The square root of this number (i.e. tau [***τ***]) is the estimated standard deviation of underlying effects across studies[25]. As with the RCT analysis, we applied a random-effects meta-analysis. Analyses were conducted using StatsDirect (version 2.5.2) and Comprehensive Meta-analysis (version 2).

## Results

### Study inclusion and methods reporting (Figure 1)

**Figure 1:**
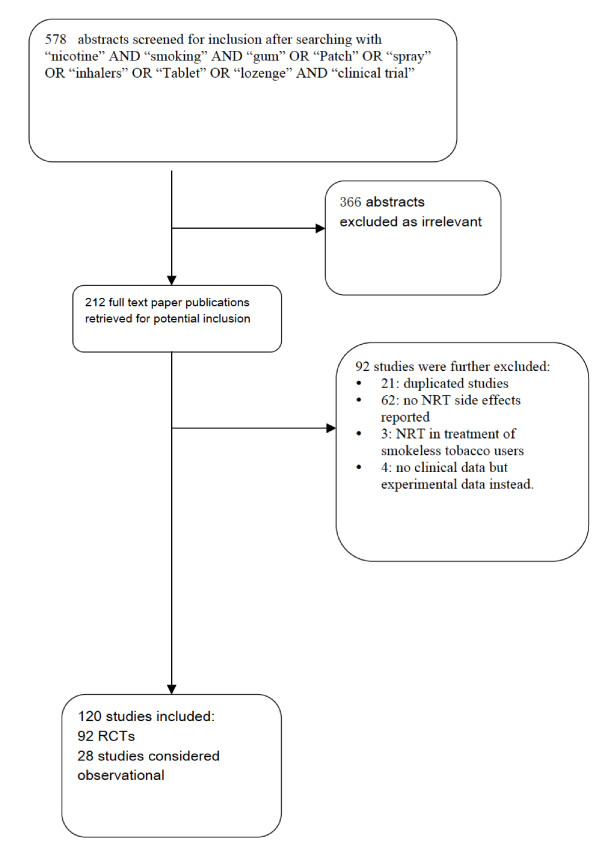
**low-diagram of included studies**.

One hundred and twenty studies met our inclusion criteria (ϕ = 0.91) [26,110-141]. Figure 1 displays the search break-down. Additional file 1 and Table 1 provide study characteristics. Ninety-two studies[26-117] were RCTs involving 32,185 participants and 28[56,75,93,110,118-141] were considered as observational studies involving 145, 205 participants.

**Table 1:** Characteristics of nicotine replacement therapy (NRT) observational studies

Author	Country	Participants	Cigarettes per day *(mean, median)	Years Smoking *(mean, median)	Intervention	Dosage (mg)	Number treated	co-intervention	Duration of treatment
Hilleman, 1994	USA	Healthy	26*	≥ 3	Patch (fixed dosage)	21	69	Education session	12 weeks
					Patch (tapered dose)	7,14,21	71		
Orleans, 1994	USA	Older adults	17*	50*	Patch	7,14,21	871	Counseling	12 weeks
Fredrickson, 1995	USA	Healthy	>20	28*	Patch	22,44	40	Counseling	8 weeks
Herrera, 1995	Sweden	Healthy	≥ 10	NA	Gum	2,4	167	Behavioural modification program	3 months
Jorenby, 1995	USA	Healthy	27*	1	Patch	22,44	504	Counseling	8 weeks
Martin, 1995	New Zealand	Healthy and unhealthy	26*	10*	Patch	7,14,21	80	Counseling	12 weeks
Smith, 1995	USA	Healthy and unhealthy	26*	20*	Patch	7,14,21	110	Counseling	12 weeks
Smith, 1996	USA	Adolescent	23*	2.6*	Patch	11,22	22	Counseling	8 weeks
Hurt, 1998	USA	Healthy	28*	22*	Nasal spray	1-2 mg/h	50	Counseling	8 weeks
Gourlay, 1999	Australia	Healthy	≥ 15	≥ 3	Patch	21	1481	Counseling	12 weeks
Hays, 1999	USA	Healthy	≥ 15	≥ 1	Patch	22	315	None	6 weeks
Killen, 1999	USA	Healthy	35*	NA	Patch	15 or 25	408	Self-treatment booklet	6 weeks
Hurt, 2000	USA	Adolescent	20*	>1	Patch	15	101	Counseling	6 weeks
Shiffman, 2002	USA	Healthy	27*	24*	Patch	7,14,21	2367	None	12 weeks
			27*	23*	Gum	2,4	2981	None	12 weeks
Croghan, 2003	USA	Healthy	≥ 15	≥ 1	Patch, Nasal spray, Patch + Nasal spray	15 mg/patch, 0.5 mg/spray	1384	Behavioural counseling	6 weeks
Hasford, 2003	Germany	Healthy and unhealthy	NA	19*	Patch	7,14,21	633	None	12 weeks
Carpenter, 2004	USA	Healthy	≥ 10	NA	Patch	7,14,21	300	Reduction counseling	6 weeks
Fiore, 2004	USA	Healthy	≥ 10	NA	Patch	7,14, 21	1869	With or without education program	10 weeks
Lerman, 2004	USA	Healthy	≥ 10	1	Patch, Nasal spray	0.5, 7-21	350	Counseling	8 weeks
Schuurmans, 2004	South Africa	Healthy	≥ 15	≥ 3	Patch	NA	184	None	10 weeks
Hughes, 2005	USA	Healthy and unhealthy	25*	27*	Nasal spray	NA	535	None	4 weeks
Marsh, 2005	Czech Republic	Medical illness	25*	≥ 1	Gum, Lozenge	4	901	None	12 weeks
Bolliger, 2007	South Africa	Healthy	23*	23*	Gum, Patch, Nasal spray	NA	100	Behavioural counseling	3 months
Aubin, 2008	USA	Healthy	23*	25*	Patch	7,14,21	370	Counseling	10 weeks
Stapleton, 2008	UK	Healthy and unhealthy	21*	NA	NRT	NA	204	Education session	12 weeks
Gunnell, 2009	Europe	Healthy and unhealthy	NA	NA	NRT	NA	63265	None	12-26 weeks
Ossip, 2009	USA	Healthy and unhealthy	NA	NA	Gum, Patch, Lozenge	2,7-21,2	65599	None	2 weeks
Steinberg, 2009	USA	Medical illnesses	>10	>20	Patch	7,14,21	64	None	10 weeks

Of RCTs, eighty-three [26-34,37,66,105-109,65-85,87-90,92-97,99-102,112,114-117,142](83/92) used a placebo control. Forty-two[39-41,43,45,47-49,51-55,60,61,64,66,68-72,74-77,81,83,87,89,90,92,93,98,99,102,103,105-107,112,115] evaluated the nicotine patch; 26[26-38,42,56,58,79,88,95,100,101,108,109,113,116,142] the nicotine gum; 6 [44,50,57,65,73,111]the nicotine nasal spray; 6[46,62,63,67,78,96] the nicotine inhaler; 4 [80,84,97,114]the nicotine tablet; 1[85] the nicotine lozenge; and, the remainder (n = 35) evaluated NRT combination therapies. Duration of treatment ranged from 1 to 24 months, with varying levels of dosages for each form of NRT.

Seventy-four RCTs[26-31,33-41,43-65,67-70,72-80,82-88,92,93,95,96,99-101,103,106-109,114-117] were conducted in healthy adult populations. An additional 6[36,66,89,97,105,113] were conducted among populations with medical and psychiatric co-morbidities (.eg. smoking-related diseases, chronic diseases, alcoholism, depression), 4[81,98,104,142] among pregnant women, 3[42,71,91] among hospitalized patients, 3[90,94,111] among adolescents, 1 [102]among postmenopausal women, and 1[112] among surgical patients. Fifty-nine [27-30,33,36-38,41,42,45,48-56,58,59,61-63,66,67,71,73,74,76,77,79,81,83,86,89-92,94,97-102,104-108,111,113-117,142]RCTs included co-interventions, of which 20[27,36,49-52,54,61,71,79,81,91,98,100-102,111,114,116,117] provided general counseling (eg. group counseling or individual counseling), 19[28,41,53,56,61,63,67,76,85,89,90,92,94,99,104-107,113] provided behavioural or psychological treatment, 12[29,30,33,37,42,45,55,62,66,74,86,97] provided varying forms of advice or support, 3[38,108,142] provided educational sessions, 4[58,59,73,77] provided an additional NRT and/or placebo, and 1[115] provided rimonabant, an endocannabinoid antagonist used as an appetite suppressant.

Seventeen observational studies[75,93,110,119-124,126-129,131-133,141] used the nicotine patch; 2 [125,135]used a nasal spray, 1[129] used the nicotine gum; and 8 used a combination of NRTs. Duration of treatment ranged from 4 to 26 weeks, with varying levels of dosages for each form of NRT. Sixteen[56,75,93,110,118,122,123,125-127,129,130,132-134,137] of the observational studies were conducted among healthy adult populations, 7 [120,121,131,135,138-140]among mixed healthy and unhealthy adult populations, 2[136,141] among adult populations with medical co-morbidities, 2[124,128] among adolescent populations, and 1[119] among an older adult population. The majority of observational studies[110,119,128,133,134,56,118,127,130,132,137,138](19/28) included co-interventions, with 12[110,119-126,128,133,134] providing general counseling; 3[56,130,137] providing counseling specific to behaviour or behaviour modification; 3[118,132,138] providing educational sessions; and 1 [127]providing a self-help booklet.

Studies reported methodological issues variably. Thirty-eight RCTs[28,29,31,37,41,44,46,59,61-63,65,70-75,77,78,81,82,84,85,90,93,97-99,101,102,104,105,112,115-117,142] reported sequence generation of randomization; 17[28,29,31,37,59,63,67,69,71,73-75,81,93,98,99,115] reported allocation concealment; and, 81[26-34,37,66,105-107,109,65-80,82-85,87-90,92-97,99-102,112,114-117,142] reported on patient blinding in the study. To confirm smoking abstinence, eighty four[26-28,30-46,48,66,105,107-109,65,67,69-80,83-98,100-104,111,112,114-117,142] studies used exhaled carbon monoxide (CO); 16[34,35,37,41,45,55,57,62,63,79,81,90,94,107,111,142] used salivary cotinine; 5[29,36,47,60,101] used serum cotinine; and 2[99,114] used urinary cotinine. No studies required participants to pay for the NRT. In 80 studies, [26-29,31-41,43-54,56,66,105-109,113,65,68,70-72,74-77,79,80,83-87,89,90,92-94,97-104,111,112-117,142] participants were planning on quitting. All observational studies are considered as non-comparative single-arm studies reporting prevalence of the adverse events in the community.

#### Adverse events

### RCTs (See Figure 2)

**Figure 2:**
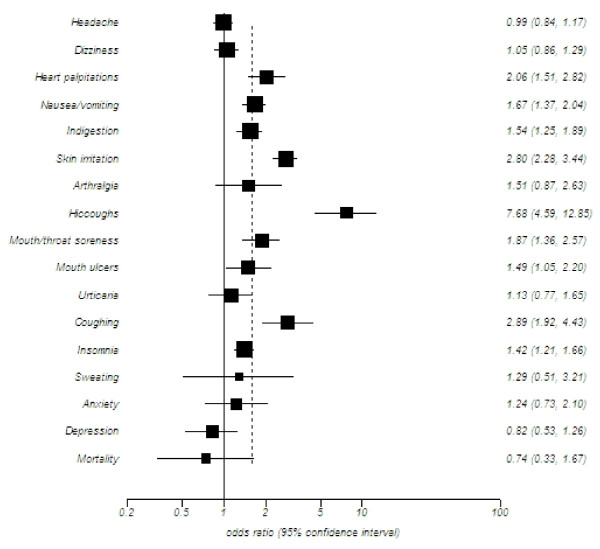
Summary pooled estimates of adverse events reported in RCTs

We evaluated all forms of NRT for adverse events. Additional file 1 displays the study characteristics and table 2 displays the RCT findings.

**Table 2:** Adverse events reported in RCTs

Adverse event	RCTs	References	*n *treatment having event	Total *n *in treatment	*n *control having event	Total *n *in control	OR	95% CI	P-value	**I**^**2**^
Headache	32	[27,33-35,41,43-45,48,50,51,55,57,58,61,64,68,71,74-76,85,90,94,95,103,115,117]	938	9227	664	5988	0.99	0.84-1.17	0.95	43%
Dizziness	24	[37-39,41,45-48,51,53,58,60,64,72,73,78,92,96,117,119]	414	4701	275	3518	1.05	0.86-1.29	0.59	18%
Heart palpitations and chest pains	12	[32,44,45,50,57,61,68,71,74,75,102,116]	189	6249	64	3985	2.06	1.51-2.82	<0.001	0%
Nausea or vomiting	31	[24,27,31,37-39,45-48,51,53,56,58,59,62,68,71,74-76,85,90,94,95,103,115]	747	7249	388	6061	1.67	1.37-2.04	<0.001	62%
Indigestion Gastrointestinal complaints	26	[27,28,32,35,37,42,43,45,51,58,63,68,70,71,80,84,85,90,94,95,105,116,117]	930	8575	489	5882	1.54	1.25-1.89	<0.001	35%
Skin irritation	32	[39-41,43,45,47,48,51,53-55,58,59,61,64,66,68-72,74-77,87,90,93,94,102,105,117]	1550	8646	511	5497	2.80	2.28-3.44	<0.001	0%
Arthralgia	5	[43,45,61,70,90]	50	756	34	686	1.51	0.87-2.63	0.13	0%
Hiccoughs	14	[27,32,34,35,37,38,63,80,83,84,94,95,117]	934	3858	573	3424	7.68	4.59-12.85	<0.001	0%
Mouth and throat soreness	23	[27,31-35,37,42,44,46,50,57,63,67,78,80,84,85,94-96,117]	935	3858	573	3424	1.87	1.36-2.57	<0.001	66%
Mouth ulcers	6	[27,33-35,63,84]	123	61	766	610	1.49	1.05-2.20	0.02	43%
Urticaria	3	[48,70,90]	80	801	66	595	1.13	0.77-1.65	0.52	0%
Coughing	12	[44,50,57,63,67,78,79,83,84,94,116]	583	3989	309	3893	2.89	1.92-4.33	<0.001	72%
Insomnia	19	[27,28,32,35,37,42,43,45,51,58,63,68,70,71,80,84,85,90,94,95,105,116,117]	636	5805	349	3504	1.42	1.21-1.66	<0.001	65%
Sweating	3	[44,50,57]	56	355	63	337	1.29	0.51-3.21	0.58	76%
Anxiety	6	[71,76,94,103,115]	25	367	21	362	1.24	0.73-2.10	0.42	0%
Depression	9	[43,45,58,64,71,94,102,103,115]	56	937	59	897	0.82	0.53-1.26	0.37	8%
Mortality	8	[73,82,85,93,97,105,113,115]	11	1387	16	1378	0.74	0.33-1.67	0.47	0%

#### Cardiovascular and respiratory

A pooled analysis of 12 RCTs found a statistically significant increased risk for heart palpitations and chest pains associated with NRT (OR 2.06, 95% CI, 1.51-2.82, p < 0.001; I^2 ^= 0%). Applying meta-regression, both the nicotine patch and orally administered NRT were associated with an increased risk (OR 1.11, 95% CI, 0.53-2.33, P = 0.75). Coughing was significantly elevated in 12 RCTs (OR 2.89, 95% CI, 1.92-4.33, P =< 0.001; I^2 ^= 72%), but was associated with considerable heterogeneity.

#### Gastrointestinal

There was a statistically significant increased risk of nausea or vomiting based on a pooled analysis of 31 RCTs (OR 1.67, 95% CI, 1.37-2.04, P =< 0.001; I^2 ^= 62%). However, meta-regression showed that studies focusing on the nicotine patch were associated with a decreased risk (OR, 0.73, 95%, 0.56-0.97, P = 0.029) of nausea and vomiting. The review also found an increased risk of indigestion and general GI complaints with NRT in an analysis of 26 RCTs (OR 1.54, 95% CI 1.25-1.89, p < 0.001; I^2 ^= 35%). Using only orally administered NRT caused significantly greater risk for GI complaints compared with the nicotine patch (OR 1.66, 95% CI, 1.04-2.63, P = 0.03), and was also associated with an increased risk of hiccoughs (see Table 2).

#### Oral

A significantly heightened risk of oral adverse events including mouth and throat soreness was identified in a meta-analysis of 23 RCTs (OR 1.87, 95% CI, 1.36-2.57, P < 0.001; I^2 ^= 66%). A meta-analysis of 6 RCTs found a significantly elevated risk for mouth ulcers (OR 1.49, 95% CI, 1.05-2.20, P = 0.02; I^2 ^= 43%).

#### Neurological

A meta-analysis of 32 RCTs (Table 2) found that headache was not associated with NRT use (P = 0.65; I^2 ^= 43%), while an analysis of 24 RCTs, found no association between NRT use and increased dizziness (P = 0.59; I^2 ^= 18%)

#### Dermatological

Nicotine patch was associated with a statistically significant risk of skin irritation in an analysis of 32 RCTs (OR 2.80, 95% CI, 2.28-3.44, P < 0.001; I^2 ^= 0%). However, nicotine patch was not associated with increased incidence of urticaria (OR 1.13, 95% CI, 0.77-1.65, P = 0.52; I^2 ^= 0%) or sweating (OR 1.29, 95% CI, 0.51-3.21, P = 0.58; I^2 ^= 76%).

#### Psychological

An increased risk of insomnia was associated with the nicotine patch (OR 1.42, 95% CI, 1.21-1.66, P < 0.001; I^2 ^= 65%). Anxiety and depression were, however, not significantly increased (see Table 2).

#### Serious Adverse Events

Twenty five RCTs[47,54,71,73-77,79,80,82,84,85,91-93,97,98,100,102,104,105,113,115,142] reported serious adverse events occurring, but none were statistically significant (data not shown). Eight studies[73,82,85,93,97,105,113,115] reported on mortality by groups and did not find a significant association between NRT and controls. One study[142] of pregnant women found two cases of spontaneous abortions in the NRT group and one study[76] reported a case of spinal meningitis in the NRT group.

### Explanations of heterogeneity

We used meta-regression to explain heterogeneity. We found large heterogeneity (I^2 ^= 62%) in our analysis of pooled events of nausea and vomiting. We were able to explain a large amount of heterogeneity examining the covariate of skin patch vs. oropharyngeal administration (OR 0.73, 95% CI, 0.56-0.97, P = 0.02, I^2 ^= 42%) as well as reporting of allocation concealment (OR 1.50, 95% CI, 1.07-1.59, P = 0.002, I^2 ^= 37%). We also found large heterogeneity (I^2 ^= 66%) in the event of mouth and throat soreness, but were unable to explain this using our pre-specified covariates. Considerable heterogeneity (I^2 ^= 72%) was also identified for the adverse event of coughing. We found that duration of study (*B *coefficient 0.07, 95% CI, 0.01-0.12, P = 0.01, I^2 ^= 42%) and allocation concealment (OR 0.53, 95% CI, 0.31-0.91, P = 0.02, I^2 ^= 43%) contributed to heterogeneity observed in this analysis. Our analysis of sweating found large heterogeneity (76%) that was predominantly contributed by a study that provided concomitant bupropion, indicating that the dual use of both drugs resulted in a significantly higher incidence of sweating (OR 29.24, 95% CI, 3.96-215.48). We also found heterogeneity in our analysis of insomnia that was explained by the duration of the trials. Longer duration trials had reduced rates of insomnia (*B *coefficient -0.07, 95% CI, -0.13 to -0.008, p = 0.02).

#### Observational studies (See Figure 3)

**Figure 3:**
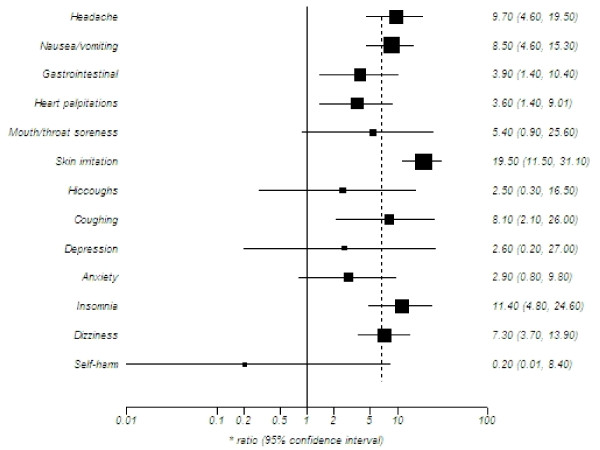
**Summary pooled estimates of adverse events reported in observational studies**.

Table 1 displays the study characteristics of the observational studies. No studies compared NRT with an inert control group, thus we present the proportions of event occurrence, interpreted as prevalence. As our analysis uses pooled proportions, all analyses found an estimate of risk, but varied in magnitude. Table 3 presents the study findings.

**Table 3:** Adverse events reported in observational studies

Adverse event	Number of studies	References	N of events	Pooled n	Proportion	95% CI	***τ***^**2**^
Headache	17	[75,93,110,118-120,123-125,127-129,131,136,138,140,141]	1472	75764	9.7	4.6-19.5	2.97
Nausea or vomiting	14	[75,110,119,120,123,124,128,129,136-138,140,141]	1902	74449	8.5	4.6-15.3	1.39
Gastrointestinal complaints	11	[110,118,120,123,127,129,131,136,138,140,141]	593	74256	3.9	1.4-10.4	3.02
Heart palpitations	7	[75,120,122,127,131,136,141]	72	2446	3.6	1.4-9.0	1.49
Mouth and throat soreness	6	[125,129,135-137,140]	406	72533	5.4	0.9-25.6	5.00
Skin irritation	16	[75,93,118-124,126-129,131,138,141]	1337	10356	19.5	11.5-31.1	1.56
Hiccoughs	4	[129,136,137,140]	375	71948	2.5	0.3-16.5	4.63
Coughing	5	[120,125,128,135,136]	125	1672	8.1	2.1-26	2.29
Depression	6	[110,120,124,138,140,141]	69	66344	2.6	0.2-27	10.57
Anxiety	5	[118,120,129,138,141]	104	5841	2.9	0.8-9.8	1.70
Insomnia	18	[75,110,118-123,125-129,131,138,140,141]	2262	76169	11.4	4.8-24.6	4.05
Dizziness	8	[110,118,120,124,127,129,136,141]	299	7338	7.3	3.7-13.9	0.94
Self-harm	1	[110,118,120,124,127,129,136,141]	141	63265	0.2	0-8.4	NA

#### Cardiovascular or respiratory

The prevalence of heart palpitations and chest pains were reported sporadically and amounted to 3.6% of the populations examined. Coughing was reported as a prevalence of 8.1%.

#### Gastrointestinal

In keeping with the RCT evidence indicating significant increases in specific events, the pooled observational evidence reported the nausea and vomiting prevalence to be 8.5% amongst individuals in the community treated with NRT. The prevalence of indigestion and other gastrointestinal complaints was 3.9%. Hiccoughs were a very common complaint in the RCT evidence, but poorly reported in the observational studies with a prevalence of just 2.5%.

#### Oral

With orally administered NRT, the prevalence of both mouth and throat soreness was 5.4%.

#### Psychological and neurological

For psychological complaints, the prevalence of insomnia was 11.4%. Anxiety and depression were more rarely reported (2.9% and 2.6% respectively). Dizziness was a more common complaint, with a prevalence of 7.3%. Headaches were commonly reported (9.7%).

#### Dermatological

The prevalence of skin irritations associated with nicotine patch was reported as 19.5% of the populations examined.

#### Serious adverse events

Serious adverse events were poorly reported in the observational studies. However, one study reported on a case of transient visual field impairment; one right-hemisphere stroke; one myocardial infraction; and one urticarial reaction from skin patch with symptoms of angiodema. Finally, in a single large observational study of self-harm (n = 63,265), 141 cases of fatal and non-fatal self-harm cases were identified (0.2%), in addition to 30 cases of suicidal ideation[139].

## Discussion

### Principal Findings

This review found that NRT is associated with an increased risk of gastrointestinal complaints and insomnia. There was also an observed increased risk of skin irritation with the nicotine patch and oropharyngeal complaints with orally administered NRT. Although NRT was associated with an increased risk of heart palpitations, the review did not observe an increased incident of heart attack or death. With the exception of insomnia, NRT does not appear to be associated with serious adverse psychiatric effects.

The reviewers actively sought serious adverse events. The most serious adverse event consistently reported in both RCTs and observational studies were heart palpitations and chest pains (OR 2.06, 95% CI, 1.51-2.82, P < 0.001). NRT has been implicated in reports of atrial fibrillation and myocardial infarction among patients with risk factors[143-148]. Several possible explanations for this exist. First, among patients using NRT who continue to smoke, high serum concentrations may stimulate the sympathetic nervous system, so increasing blood pressure, stroke volume and cardiac output[149]. Second, previous and current smokers may have established cardiovascular disease. Patients with unstable coronary syndrome, a common manifestation of coronary artery disease, may have unrecognized recent plaque ruptures including coronary vasoconstriction and increased strain placed on the heart due to tachycardia and hypertension[143]. Unofficial guidelines[149] caution the continued use of NRT in patients with known cardiovascular disease in the absence of a physician. They recommend that patients be warned of these risks and counseled to desist smoking and arrange intensive behavioral support[149]. In our analysis, we did not observe an increased risk for myocardial infarction or death from NRT.

Almost all studies demonstrated localized irritation related to NRT use, skin irritation with the use of NRT patch and mouth soreness and ulcerations with orally administered NRT. It is possible this is due to the success of cessation rather than NRT as mouth ulcers occur in about 40% of all individuals achieving tobacco abstinence regardless of cessation intervention[150]. It has previously been understood that mouth lesions are associated with smoking cessation and not NRT[150]. However, this review found a significantly increased risk of mouth ulcers with orally administered NRT users compared to inert controls who had ceased smoking. In order to prevent relapse due to treatment discontinuation, strategies should be developed to assist patients unable to continue oral administered NRT due to mouth ulcers such as increasing the nicotine patch dose, using the nicotine nasal spray, or switching to an alternative form of smoking cessation pharmacotherapy such as buproprion or varenicline.

Psychological adverse events, particularly suicidal ideation, are a major concern in patients initiating smoking cessation[151]. We found only one large retrospective observational study that discussed this topic and reported no significant difference in fatal and non-fatal self-harm associated with NRT compared to other frequently used pharmacotherapies, bupropion (HR 1.17, 95% CI, 0.59-2.32) or varenicline (HR 1.12, 95% CI, 0.67-1.88)[139].

A criticism of smoking cessation trials has been that they infrequently enroll participants with psychological difficulties, thus making generalizable statements about their safety difficult[152]. The present review found that 56 RCTs specifically excluded participants with mental disorders. Only two RCTs targeting participants with concomitant psychological difficulties, specifically alcoholism and depression, were identified[89,92]. In these studies, the risk of insomnia was higher among those taking NRT, compared to controls, (OR 1.42, 95% CI, 1.21-1.66, P < 0.001). Sleep disturbance can significantly worsen psychological distress and mental illness and impair quality of life[153]. Therefore, clinicians should remain vigilant about NRT-related sleep disturbance among patients with a history of psychiatric illness.

An important issue to examine when describing adverse events from smoking cessation therapies is whether the adverse events are related to a pharmacotherapy or whether they are related to tobacco withdrawal[154]. For example, insomnia and sleep disturbances could be related to tobacco abstinence. One way to assess this affect would be to compare side effects in those that have quit smoking in both groups. However, since individuals that quit smoking may differ from those that continue, this analysis would remove the benefits of randomization and introduce bias.

## Limitations

Our review has several limitations. These include limitations of the primary studies themselves as well as those associated with combining results across potentially heterogeneous studies or populations. The main limitation of the primary studies is the mechanism by which adverse events are recorded. In the majority of instances this would be through passive reporting and therefore be susceptible to the underreporting associated with such techniques. The majority of our analyses had low or moderate heterogeneity, although a few had high levels of heterogeneity. Pooling proportions always results in large estimates of heterogeneity and statistical techniques do not yet exist to interpret the extent of real between-study heterogeneity[155]. The review identified some discrepancies between observational studies and RCTs in terms of adverse event reporting. Possible explanations of this include the use of a control group in the RCTs, which diminishes the impact of adverse events that are, in fact, unrelated to the intervention. Studies included in our review varied in the duration of treatment phase. While we would expect most adverse events to occur during the treatment phase (receiving active NRT), it is possible that some adverse events occurred during follow-up and we cannot adequately explain their pathological processes.

There is emerging evidence that stopping smoking prior to any type of surgery decreases the potential for surgical complications[156]. All pharmacotherapies used for smoking cessation demonstrate adverse events, albeit in differing conditions and severity[4]. However, given the cardiovascular concerns discussed above, and the fact that cardiovascular events are increased during the perioperative period[157], it is reasonable to consider other behavioral or pharmacotherapies for at-risk patients undergoing major surgeries.

## Conclusions

This review demonstrates that NRT is associated with adverse effects that may be discomforting for the patient but are not life-threatening. Given the long-term benefits of smoking cessation over continued smoking, concern about NRT related adverse events should be balanced against the benefits of cessation. Clinicians should monitor for side effects that may worsen underlying conditions, such as insomnia in patients with depression, and consider additional or alternative treatments. Given the benefits of smoking cessation and the important role of NRT in achieving this goal, efforts should be made to counsel patients on the most common side effects and strategies should be developed to deal with them.

## Competing interests

Pfizer Ltd. Walton Oaks, Walton-On-The-Hill, Surrey, KT20 7NS, United Kingdom, sponsored development of the review and manuscript. Edward Mills and Ping Wu were paid consultants to Pfizer in connection with the development of this manuscript. Kumanan Wilson and Job O. Ebbert received no compensation. Ian Lockhart is an employee of Pfizer.

## Authors' contributions

EM, PW, IL, KW conceived the study. EM, PW carried out searches and data abstraction. EM, PW, IL, KW, JE were involved in data analysis. EM, PW, IL, KW, JE contributed to manuscript drafting and interpretation of data. All authors read and approved the final draft.

## Supplementary Material

Additional file 1 **Characteristics of included RCTs**. CVD, cardiovascular; RCT, randomized clinical trial Click here for file
