# Supplementary material for: Adverse events associated with nicotine replacement therapy (NRT) for smoking cessation. A systematic review and meta-analysis of one hundred and twenty studies involving 177,390 individuals
Source: Tob Induc Dis. 2010 Jul 13;8(1):8. doi: 10.1186/1617-9625-8-8 (PMC2917405; doi:10.1186/1617-9625-8-8)
Supplement: Additional file 1 — Characteristics of included RCTs. CVD, cardiovascular; RCT, randomized clinical trial [file 1617-9625-8-8-S1.DOC]

| **Author** | **Country** | **Participants** | **Cigarettes per day *(mean, median)** | **Years**  **Smoking *(mean, median)** | **Intervention** | **Dosage (mg)** | **Treatment and number (n) in each group** | | | | **Co-intervention** | **Duration of treatment** |
| --- | --- | --- | --- | --- | --- | --- | --- | --- | --- | --- | --- | --- |
|  |  |  |  |  |  |  | **Intervention** | **n** | **Control** | **n** |  |  |
| Malcolm, 1980 | England | Healthy | 25* | NA | Gum | 2 | Gum | 73 | Placebo | 63 | None | 3 months |
| Fagerstrom, 1982 | Sweden | Healthy | NA | NA | Gum | 2 | Gum | 47 | Placebo | 49 | Psychological treatment | 4 weeks |
| Jarvis, 1982 | England | Healthy | >20 | NA | Gum | 2 | Gum | 58 | Placebo | 58 | Group counseling | 12 months |
| British Thoracic Society, 1983 | England | Healthy | ≥1 | NA | Gum | 2 | Gum | 410 | Placebo | 412 | Verbal advice + booklet | 3-6 months |
|  |  |  |  |  |  |  |  |  | No treatment | 401 |  |  |
| Schneider, 1983 | USA | Healthy | ≥20 | NA | Gum | 2 | Gum | 30 | Placebo | 30 | Clinic-support | NA |
|  |  |  |  |  |  |  |  | 13 |  | 23 | Minimal intervention |  |
| Jamrozik, 1984 | England | Healthy | NA | NA | Gum | 2 | Gum | 101 | Placebo | 99 | None | 3 months |
| Areechon, 1988 | Sweden | Healthy | ≥15 | 24* | Gum | 2 | Gum | 98 | Placebo | 101 | Lecture | 3 months |
| Fortmann, 1988 | USA | Healthy | 24* | 25* | Gum | 2 | Gum | 299 | Placebo | 148 | None | 3 months |
| Harackiewicz, 1988 | USA | Healthy | 26.5* | 17* | Gum | 2 | Gum | 99 | Self-help manual | 52 | None | 1.5-6 months |
|  |  |  |  |  |  |  |  |  | Short booklet | 46 |  |  |
| Tonnesen, 1988 | Sweden | Healthy with Chronic Disease | ≥10 | NA | Gum | 2 | Gum | 60 | Placebo | 53 | Counseling | 2-24 months |
| Tonnesen, 1988 | Denmark | Healthy | ≥10 | 10-65 | Gum | 2,4 | Gum | 116 | Advice | 56 | None | ≥6 weeks |
| Abelin, 1989 | Switzerland | Healthy | >20 | 21* | Patch | 7 to 21 | Patch | 100 | Placebo | 99 | None | 12 weeks |
|  |  |  |  |  |  |  |  | 56 |  | 56 |  |  |
| Blondal, 1989 | Iceland | Healthy | 21g* | NA | Gum | 4 | Gum | 92 | Placebo | 90 | Education session | 3 months |
| Hughes, 1989 | USA | Healthy | 30* | 19* | Gum | 2 | Gum | 210 | Placebo | 105 | Brief advice | 3 months |
| Hughes, 1990 | USA | Healthy | 30* | 19* | Gum | 0.5-4 | Gum | 59 | Placebo | 19 | None | 9 months |
| Hurt, 1990 | USA | Healthy | ≥20 | ≥1 | Patch | 21 | Patch | 35 | Placebo | 35 | None | 6 weeks |
| Jensen, 1990 | Denmark | Healthy | >10 | 5 | Gum | 2 | Gum | 211 | Placebo | 82 | Education meeting | 6-12 weeks |
| Campbell, 1991 | England | Hospitalized patients | NA | NA | Gum | 4 | Gum | 107 | Placebo | 105 | Advice | 3-12 months |
| Daughton, 1991 | USA | Healthy | ≥20 | 23.9* | Patch | NA | Patch (24 hour) | 51 | Placebo | 52 | None | 4 weeks |
|  |  |  |  |  |  |  | Patch (wakeful hour) | 55 |  |  |  |  |
| Tonnesen, 1991 | Sweden | Healthy | ≥10 | ≥3 | Patch | 15 | Patch | 145 | Placebo | 144 | Psychological support | 12 weeks |
| Transdermal Nicotine study group, 1991 | USA | Healthy | 31* | 24* | Patch | 7,14,21 | Patch | 664 | Placebo | 271 | Behavioural therapy | 6 weeks |
| Sutherland, 1992 | England | Healthy | 25* | 22* | Nasal spray | 1 | Spray | 116 | Placebo | 111 | None | 3 months |
| Merz, 1993 | Germany | Healthy | 27* | 18* | Patch | 7 to 21 | Patch | 80 | Placebo | 80 | None | 3 months |
| Russell, 1993 | 15 English counties | Healthy | ≥15 | NA | Patch | 5 to 15 | Patch | 400 | Placebo | 200 | Brief support | 18 weeks |
| Sachs, 1993 | USA | Healthy | ≥10 | ≥3 | Patch | 15 | Patch | 113 | Placebo | 107 | Physician counseling | 12-18 weeks |
| Tonnesen, 1993 | Sweden | Healthy | ≥10 | ≥3 | Inhalers | 0.1umol/  per puff | Inhaler | 145 | Placebo | 141 | None | 3-6 months |
| Westman, 1993 | USA | Healthy | 30* | 22* | Patch | 12.5 to 25 | Patch | 79 | Placebo | 80 | Counseling | 6 weeks |
| Fiore, 1994 | Ireland | Healthy | ≥15 | ≥1 | Patch | 22 | Patch | 44 | Placebo | 43 | Group counseling | 8 weeks |
|  |  |  |  |  |  | 11 to 22 |  | 57 |  | 55 | Individual counseling | 6 weeks |
| Hjalmarson, 1994 | Sweden | Healthy | 21* | 26* | Nasal spray | 1 | Spray | 125 | Placebo | 123 | Group counseling | 3 months |
| Hurt, 1994 | USA | Healthy | ≥20 | Past 1 year | Patch | 22 | Patch | 120 | Placebo | 120 | Individual counseling | 8 weeks |
| Levin, 1994 | USA | Healthy | 28* | 23* | Patch | 22 | Patch | 31 | Placebo | 31 | Group counseling | 8 weeks |
| Richmond, 1994 | Australia | Healthy | 29* | 24* | Patch | 7 to 21 | Patch | 158 | Placebo | 157 | Behavioural therapy | 10 weeks |
| Dale, 1995 | USA | Healthy | ≥10 | ≥1 | Patch | 11 | Patch | 18 | Placebo | 18 | None | 8 weeks |
|  |  |  |  |  |  | 22 |  | 17 |  |  |  |  |
|  |  |  |  |  |  | 44 |  | 18 |  |  |  |  |
| Gourlay, 1995 | Australia | Healthy | 32* | 23* | Patch | 7 to 21 | Patch | 315 | Placebo | 314 | Behavioural counseling | 3 months |
| Herrera, 1995 | Sweden | Healthy | ≥10 | NA | Gum | 2 | Gum | 76 | Placebo | 78 | Behavioural modification program | 3 months |
| Kornitzer, 1995 | Sweden | Healthy | 25* | 22* | Gum | 2 | Gum | 149 | Placebo | 150 | Patch | 12-24 weeks |
|  |  |  |  |  | Patch | 5 to 15 | Patch | 150 | Placebo | 75 | Placebo gum |  |
| Puska, 1995 | Finland | Healthy | 21* | 21* | Patch | 15 | Patch | 150 | Placebo | 150 | Gum | 12-18 weeks |
| Schneider, 1995 | USA | Healthy | ≥15 | 22* | Nasal spray | 1 | Spray | 128 | Placebo | 127 | None | 6 weeks - 6 months |
| Stapleton, 1995 | England | Healthy | ≥15 | NA | Patch | 15 | Patch | 400 | Placebo | 400 | Booklet + advice | 18 weeks |
|  |  |  |  |  |  | 25 |  | 400 |  |  |  |  |
| Campbell, 1996 | England | Smoking-related disease | ≥1 | Past 1 week | Patch | 7 to 21 | Patch | 115 | Placebo | 119 | Advice | 12 weeks |
| Joseph, 1996 | USA | Cardiac disease | 28* | 44* | Patch | 7,14,21 | Patch | 294 | 290 | 60 | Behavioural counseling | 10 weeks |
| Leischow, 1996 | USA | Healthy | 26* | 25* | Inhalers | NA | Inhaler | 111 | Placebo | 111 | Advice | 3-6 months |
| Murray, 1996 | Canada | Chronic obstructive pulmonary disease | 28* | NA | Gum | 2 | Gum | 3923 | No treatment | 1964 | Behavioural therapy | 4 months |
| Paoletti, 1996 | Italy | Healthy | 23* | 21* | Patch | 15 | Patch | 60 | Placebo | 60 | None | 12 weeks |
| Schneider, 1996 | USA | Healthy | 26* | 25* | Inhalers | 13ug/ per puff | Inhaler | 112 | Placebo | 111 | Behavioural intervention | 6 months |
| Blondal, 1997 | Sweden | Healthy | ≥1 | 2.7 | Nasal spray | 1 | Spray | 79 | Placebo | 78 | None | 3 months |
| Hjalmarson, 1997 | USA | Healthy | ≥10 | ≥3 | Inhalers | 13 ng/ puff | Inhaler | 123 | Placebo | 124 | Behavioural modification program | 3-6 months |
| Sonderskov, 1997 | Denmark | Healthy | <20 | NA | Patch | 14 | Patch | 119 | Placebo | 125 | None | 12 weeks |
|  |  |  | ≥20 |  |  | 21 |  | 132 |  | 142 |  |  |
| Ahluwalia, 1998 | USA | Healthy | ≥10 | >1 | Patch | 7 to 21 | Patch | 205 | Placebo | 205 | None | 10 weeks |
| Buchkremer, 1998 | Germany | Healthy | 29* | NA | Patch | NA | Patch | 43 | Placebo | 42 | Behavioural therapy | 6 weeks |
| Davidson, 1998 | USA | Healthy | ≥20 | ≥1 | Patch | 30 | Patch | 401 | Placebo | 401 | None | 6 weeks |
| Lewis, 1998 | USA | Hospitalized patients | 24* | 27* | Patch | 11 to 22 | Patch | 62 | Placebo | 62 | Counseling | 6 weeks |
| Perng, 1998 | Taiwan | Healthy | ≥20 | 33* | Patch | 30 | Patch | 30 | Placebo | 32 | None | 6 weeks |
| Blondal, 1999 | Sweden | Healthy | 25* | ≥3 | Nasal spray | 0.5 | Spray | 120 | Placebo | 119 | Nicotine patch | 1 year |
| Hays, 1999 | USA | Healthy | ≥15 | ≥1 | Patch | 22 | Patch | 321 | Placebo | 322 | None | 6 weeks |
| Jorenby, 1999 | USA | Healthy | 25* | 25* | Patch | 7 to 21 | Patch | 244 | Placebo | 160 | Behavioural treatment | 8 weeks |
|  |  |  |  |  |  |  | Patch + Bupropion | 245 | Placebo + Burpropion | 244 |  |  |
| Tonnesen, 1999 | Europe | Healthy | ≥14 | ≥3 | Patch | 15 | Patch | 716 | Placebo | 714 | Advice brochure | 8 weeks |
|  |  |  |  |  |  |  |  | 715 |  |  |  | 22 weeks |
|  |  |  |  |  |  | 25 | Patch | 715 |  |  |  | 8 weeks |
|  |  |  |  |  |  |  |  | 715 |  |  |  | 22 weeks |
| Bolliger, 2000 | Switzerland | Healthy | ≥15 | ≥3 | Inhalers | 13ug/  per puff | Inhaler | 200 | Placebo | 200 | None | 4-18 months |
| Bohadana, 2000 | France | Healthy | ≥10 | ≥3 | Patch | 15 | Patch | 200 | Placebo | 200 | Nicotine inhaler | 6 weeks |
| Garvey, 2000 | USA | Healthy | 5 | NA | Gum | 2 | Gum | 202 | Placebo | 203 | Counseling | 2 months |
|  |  |  |  |  |  | 4 | Gum | 203 |  |  |  |  |
| Wisborg, 2000 | Denmark | Pregnant women (>22 weeks) | ≥10 | NA | Patch | 10 to 15 | Patch | 124 | Placebo | 126 | Counseling | 11 weeks |
| Wallstrom, 2000 | Sweden | Healthy | 19* | 26* | Tablet | 2 | Tablet | 123 | Placebo | 124 | None | 3-6 months |
| Etter, 2002 | Switzerland | Healthy | ≥20 | 3 | Gum, Patch, Nasal spray | 2,15,0.5 | NRT | 265 | Placebo | 269 | None | 6 months |
|  |  |  |  |  |  |  |  |  | No treatment | 389 |  |  |
| Glover, 2002 | USA | Healthy | ≥10 | ≥ 3 | Tablet | 2 | Tablet | 120 | Placebo | 121 | None | 3-6 months |
| Hand, 2002 | England | Smoking-related disease | ≥1 | NA | Patch, Inhalers | 10 to 30 | NRT | 136 | No treatment | 109 | Advice and support | 3 weeks |
| Shiffman, 2002 | USA, England | Healthy TTFC<30min | 17* | NA | Lozenge | 2 | Lozenge | 459 | Placebo | 458 | Behavioural support | 6 months |
|  |  | Healthy TTFC>30min |  |  |  | 4 |  | 450 |  | 451 |  |  |
| Shiffman, 2002 | USA | Healthy | 25* | 24* | Patch | 7 to 21 | Patch | 283 | Placebo | 284 | None | 10 weeks |
| Glavas, 2003 | Croatia | Healthy | ≥1 | ≥1 | Patch | 7 to 21 | Patch | 56 | Placebo | 56 | None | 3 weeks |
| Hanson, 2003 | USA | Adolescent | ≥15 | >0.5 | Patch | 7 to 21 | Patch | 50 | Placebo | 50 | Cognitive behavioural therapy | 10 weeks |
| Hughes, 2003 | USA | Alcoholism | ≥20 | NA | Patch | 7 to 21 | Patch | 61 | Placebo | 54 | Behavioural therapy | 12 weeks |
| Molyneux, 2003 | England | Hospitalized patients | 20* | 33* | Gum, Patch, Nasal spray, Inhalers, Tablet, Lozenge | 2,15,0.5,10,2 | NRT | 91 | No treatment | 91 | Counseling | 6 weeks |
| Smith, 2003 | USA | Healthy with depression | ≥15 | ≥1 | Patch | 7 to 21 | Patch | 244 | Placebo | 160 | Behavioural treatment | 8 weeks |
|  |  |  |  |  |  |  | Patch + Bupropion | 245 | Placebo + Burpropion | 244 |  |  |
| Wennike, 2003 | Sweden | Healthy | ≥15 | ≥3 | Gum | 2 | Gum | 65 | Placebo | 68 | None | 12 months |
|  |  |  |  |  |  | 4 | Gum | 140 | Placebo | 138 |  |  |
| Schuurmans, 2004 | South Africa | Healthy | ≥15 | ≥3 | Patch | NA | Patch | 100 | Placebo | 100 | None | 2 weeks |
| Batra, 2005 | Europe | Healthy | ≥20 | ≥3 | Gum | 4 | Gum | 184 | Placebo | 180 | None | 12 months |
| Moolchan, 2005 | Canada | Adolescent | ≥10 | ≥0.5 | Gum | 2 to 4 | Gum | 46 | Placebo | 40 | Cognitive behavioural therapy | 12 weeks |
|  |  |  |  |  | Patch | 14 to 21 | Patch | 34 |  |  |  |  |
| Warner, 2005 | USA | Surgery | ≥10 | Past 30 days | Patch | 21,42 | Patch | 62 | Placebo | 59 | None | 4 weeks |
| Hotham, 2006 | Australia | Pregnant (12-28 weeks) | ≥15 | NA | Gum | 15 | Patch | 20 | No treatment | 20 | Counseling | 12 weeks |
| Rennard, 2006 | USA | Healthy | ≥20 | ≥3 | Inhalers | 10 | Inhaler | 215 | Placebo | 214 | None | 12 months |
| Tonnesen, 2006 | Sweden | Chronic obstructive pulmonary disease | 20* | NA | Tablet | 2 | Tablet | 95 | Placebo | 88 | Low support | 12 weeks |
|  |  |  |  |  |  |  |  | 90 |  | 97 | High support |  |
| Covey, 2007 | USA | Healthy | ≥10 | NA | Patch | 7 to 21 | Gum + Bupropion | 74 | Placebo + Bupropion | 74 | Counseling | 16 weeks |
|  |  |  |  |  |  |  | gum | 73 | Placebo | 73 |  |  |
| Myung, 2007 | Korea | Healthy | 15* | 16.5* | Patch | 7 to 21 | Patch | 59 | Placebo | 59 | Behavioral counseling | 6 weeks |
| Oncken, 2007 | USA | Post-menopausal women | ≥10 | 33* | Patch | 21 | Patch | 57 | Placebo | 95 | Group counseling | 12 weeks |
| Piper, 2007 | USA | Healthy | ≥10 | NA | Gum | 2 | Gum + Bupropion | 228 | Placebo + Bupropion | 224 | Counseling | 9 weeks |
| Pollak, 2007 | USA | Pregnant women (12-25 weeks) | ≥100 (lifetime) | NA | Gum, Patch, Lozenge | 2,15,0.5,10,2 | NRT | 122 | No treatment | 59 | Behavioral therapy | 6 weeks |
| Uyar, 2007 | Turkey | Healthy | ≥10 | >1 | Patch | 7 to 21 | Patch | 50 | Advice | 31 | None | 6 weeks |
| Oncken, 2008 | USA | Pregnant women | 10* | NA | Gum | 2 | Gum | 100 | Placebo | 94 | Education meeting | 6-12 weeks |
| Rubinstein, 2008 | USA | Adolescent | >5 | >6 months | Nasal spray | 0.5 | Spray | 23 | No treatment | 17 | Counseling | 8 weeks |
| Piper, 2009 | USA | Healthy | 21* | ≥6 months | Patch | 7,14,21 | Patch | 262 | Placebo | 189 | Counseling | 12 weeks |
|  |  |  |  |  | Lozenge | 2,4 | Lozenge | 260 |  |  |  |  |
|  |  |  |  |  | Patch + Lozenge | 7,14,21; 2,4 | Patch + Lozenge | 267 |  |  |  |  |
| Rigotti, 2009 | USA | Healthy | 23* | 25* | Patch | 7,14,21 | Patch | 369 | Placebo | 366 | Rimonabant (20 mg daily) | 10 weeks |
| Shiffman, 2009 | USA | Healthy | NA | 26* | Gum | 2,4 | Gum | 1649 | Placebo | 1648 | Counseling | 12-24 weeks |
| Sun, 2009 | China | Healthy | 23* | 21* | Tablet | 2 | Tablet | 101 | Placebo | 110 | Counseling | 2 months |
